# When schools were open for in-person teaching during the COVID-19 pandemic - the nordic experience on control measures and transmission in schools during the delta wave

**DOI:** 10.1186/s12889-022-14906-y

**Published:** 2023-01-09

**Authors:** Torill Alise Rotevatn, Karin Nygård, Laura Espenhain, Rebecca Legarth, Karina Lauenborg Møller, Emmi Sarvikivi, Otto Helve, Guðrún Aspelund, Annika Ersson, Marie Nordahl, Margrethe Greve-Isdahl, Elisabeth Astrup, Tone Bjordal Johansen

**Affiliations:** 1 Norwegian Institute of Public Health, Oslo, Norway; 2 Statens Serum Institut, Copenhagen, Denmark; 3 Finnish Institute for Health and Welfare, Helsinki, Finland; 4 The Directorate of Health, Reykjavik, Iceland; 5 The Public Health Agency of Sweden, Stockholm, Sweden

**Keywords:** SARS-CoV-2, COVID-19, Schools, Incidence trends, Vaccination, Mitigation measures

## Abstract

**Background:**

Extensive measures to control spread of SARS-CoV-2 have led to limited access to education for millions of children and adolescents during the COVID-19 pandemic. Education and access to schools is vital for children and adolescents’ learning, health, and wellbeing. Based on high vaccine uptake and low incidence levels, the Nordic countries (Denmark, Finland, Iceland, Norway and Sweden) decided to start the academic year 2021/22 with schools open for in-person teaching and moderate mitigation measures. We describe trends in SARS-CoV-2 infections and vaccination coverage among students during the first 12 weeks of the fall semester.

**Methods:**

In this multinational, retrospective, observational study, we have used surveillance and registry data from each of the Nordic countries to describe vaccine uptake (≥12 years), infection incidence (whole population) and transmission of SARS-CoV-2 among students. The study period, week 30 to 41 (Jul 26th – Oct 17th), represents the autumn semester from immediately before school started until fall break. In addition, we collected information on mitigation measures applied by the respective countries.

**Results:**

There were slight variations between the countries regarding existing infection prevention and control (IPC) measures, testing strategies and vaccination start-up among adolescents. All countries had high vaccine uptake in the adult population, while uptake varied more in the younger age groups. Incidence in the school-aged population differed between countries and seemed to be influenced by both vaccine uptake and test activity. Infection clusters among school-aged children were described for Denmark and Norway, and the number of clusters per week reflected the incidence trend of the country. Most events consisted of only 1–2 cases. Larger clusters appeared more frequently in the higher grades in Norway and in lower grades in Denmark.

**Conclusion:**

Data from the Nordic countries indicate that vaccination of adults and adolescents, in addition to mitigation measures, enabled full in-person learning. As SARS-CoV-2 infection does not represent a severe medical risk for most children as previously thought, measures targeting this group should be carefully adjusted and kept at a minimum. Our data add to the evidence on incidence and transmission of SARS-CoV-2 among students in schools open for in-person teaching, and may be valuable for decision makers worldwide.

## Background

Transmission of SARS-CoV-2 in schools has been a prevailing concern during the pandemic, resulting in extensive measures in schools and limited access to education for millions of children and adolescents [1]. School closure has been frequently used to prevent in-school transmission, although children and adolescents have very low risk for severe disease from SARS-CoV-2 infection [2–6]. School closures have profound negative consequences for students’ learning, wellbeing and mental health [7–9], and has not been found more effective to reduce COVID-19 transmission than in-person teaching with mitigation measures [10–14]. As an alternative to full closure, hybrid teaching has been frequently used to allow implementation of contact reducing and distancing measures in schools. Although less disruptive than full closure, these measures are also associated with negative effects on learning and well-being for students [15–25]. Taken together, the low risk for severe disease in these age-groups, the questionable effectiveness of school closures and the negative consequences of strict infection prevention and control (IPC) measures underline the urgency of securing a normal educational and social life for children and adolescents.

The SARS-CoV-2 Delta variant became dominant in the Nordic countries (Denmark, Finland, Iceland, Norway and Sweden) over the summer of 2021. This variant showed increased transmissibility compared with previous variants, and there were concerns about reduced vaccine effectiveness [26]. Altogether, these virus properties led to growing concern about transmission in schools. After the summer, however, the vaccination coverage in the adult population was high and incidence levels were low [27–31]. Thus, following nearly 1.5 years of varying, but overall strict interventions in schools, the Nordic countries started the academic year 2021/22 with schools open for full in-person teaching and only moderate mitigation measures [27–31]. The measures, with slight variations between countries, included stay-home-when-sick policy, hygiene measures, testing of close contacts, isolation of positive cases, limited use of quarantine for school contacts, and very limited use of distance measures and face masks. The decision to offer full in-person teaching was additionally based on the low severity of disease in children [2–6] and on evidence showing that transmission in schools was limited [10, 32–34]. Furthermore, all the Nordic countries initiated vaccination of adolescents before or around start of the semester, but timing of initiation and implementation in different age groups varied between the countries.

We here share experience from the Nordic countries where schools were kept open for in-person teaching in a period when many other countries still had stricter measures and more limited access to school [35]. The Nordic countries are similar in many ways, and they have comparable infection surveillance systems that provide a good overview of the epidemiological situation. The study period represented a window when measures in schools and vaccine uptake in adolescents differed slightly between the countries, while vaccination coverage among adults was high. Our aim was to describe trends in reported SARS-CoV-2 infections and vaccination coverage among students in the context of infection rates and vaccination uptake in the general population and school-specific IPC measures. We also describe infection clusters in Danish and Norwegian schools during the same period.

## Methods

### Study design and setting

We performed a multinational, retrospective observational study during a period when the SARS-CoV-2 Delta variant dominated [36]. The study was conducted as a collaboration between the national public health institutes in Denmark, Finland, Iceland, Norway and Sweden.

The study period was set to weeks 30 to 41 (Jul 26th – Oct 17th) 2021. In the Nordic countries, schools resume after summer earlier than in most European countries (time for first day in school after summer varied between Aug 9th – 29th) and the study period thus represents the time from immediately before school started and until autumn break. The schools were open for in-person teaching with moderate mitigation measures in all the Nordic countries.

### Study population and data sources

We included the total population of all five countries and categorized the populations into age groups according to initiation of vaccination: 6–11 years, 12–15 years, 16–17 years and ≥ 18 years.

We obtained data on weekly number of COVID-19 cases for the whole population and vaccination coverage for the population aged 12 years and up. Data originated from each country’s national database on COVID-19 cases and vaccination registries (Table 1). All reported SARS-CoV-2 infections were PCR-confirmed during the study period. To identify clustering of cases in schools we obtained data on school affiliation from national education registries. Furthermore, we collected country-specific information on population sizes and on existing IPC measures in schools, as well as testing and vaccination strategies for school-aged children and adolescents.

**Table 1:** Data sources applied by all participating countries

Type of information	Denmark	Finland	Iceland	Norway	Sweden
**Collaborative institution**	Statens Serum Institut https://en.ssi.dk/	Finnish Institute for Health and Welfare https://thl.fi/en/web/thlfi-en	The Directorate of Health https://www.landlaeknir.is/english/	Norwegian Institute of Public Health https://www.fhi.no/en/	The Public Health Agency of Sweden https://www.folkhalsomyndigheten.se/the-public-health-agency-of-sweden/
**COVID-19 cases**	The Danish Microbiology database https://miba.ssi.dk/service/english	The National Infectious Disease Registry https://thl.fi/en/web/infectious-diseases-and-vaccinations/surveillance-and-registers/finnish-national-infectious-diseases-register	The Registry of Communicable Diseases https://www.landlaeknir.is/tolfraedi-og-rannsoknir/gagnasofn/gagnasafn/item35278/Smitsjukdomaskra-(Register-of-Communicable-Diseases)	The Norwegian Surveillance System for Communicable Diseases (MSIS) https://www.fhi.no/en/hn/health-registries/msis/	The Swedish Surveillance System for Communicable Diseases (Sminet) https://www.folkhalsomyndigheten.se/the-public-health-agency-of-sweden/communicable-disease-control/surveillance-of-communicable-diseases/
**Vaccination coverage**	The Danish Vaccination Registry https://www.ssi.dk/vaccinationer/boernevaccination/vaccinationsdaekning-og-aarsraporter/det-danske-vaccinationsregister-ddv	The National Vaccination Registry https://thl.fi/en/web/infectious-diseases-and-vaccinations/surveillance-and-registers/finnish-national-vaccination-register-and-monitoring-of-the-vaccination-programme	The Vaccination Registry https://www.landlaeknir.is/tolfraedi-og-rannsoknir/gagnasofn/gagnasafn/item15627/Bolusetningaskra-(Vaccination-Register)	The Norwegian Immunisation Registry (SYSVAK) https://www.fhi.no/en/hn/health-registries/norwegian-immunisation-registry-sysvak/	The National Vaccination Register (NVR) https://www.folkhalsomyndigheten.se/the-public-health-agency-of-sweden/communicable-disease-control/vaccinations/vaccination-register-and-vaccination-coverage/variable-list-for-the-national-vaccination-register/
**Population**	Statistics Denmark https://www.dst.dk/en	The Population Registry, Statistics Finland https://www.stat.fi/en/statistics/vaerak	The National Statistical Institute of Iceland https://www.statice.is/statistics/population/inhabitants/	The National Population Register https://www.skatteetaten.no/en/person/national-registry/	Statistics Sweden (SCB) https://www.scb.se/en/finding-statistics/statistics-by-subject-area/population/population-composition/population-statistics/
**Educational data**	The Ministry of Health’s student register https://www.dst.dk/en/Statistik/dokumentation/documentationofstatistics/the-student-registre			The National Education Database https://www.ssb.no/a/english/mikrodata/datasamling/nudb/nudb_20130607-en.html	

### COVID-19 school clusters

We included data on school clusters of COVID-19 to study trends in cluster size in the context of community transmission rates. This was restricted to the countries that had established surveillance systems to identify clusters in schools at the time of data collection (Denmark and Norway). These data give an indication of transmission and outbreaks that may have occurred in schools. Clusters were identified by linking data on cases with data on school affiliation and birth year [34]. Cases were clustered together if they occurred at the same school and in the same age cohort within a period of 14 days. Fourteen days without new cases marked the end of a cluster, whereafter a new cluster could start.

### Analysis

To make incidence rates comparable across countries and age groups, we calculated country-specific incidence per 100.000 (cases*(100.000/population)), per week for the age groups 0–5 years, 6–11 years, 12–15 years, 16–17 years and ≥ 18 years. Weekly incidence rates were then plotted against weekly vaccination coverage to illustrate differences in trends in infection rates and vaccination coverage in different age groups across countries. Finally, we plotted number of clusters stratified by cluster size (1–2, 3–5, 6–9, 10–19 and 20+ cases) against incidence rates in the total population in order to study trends in infection clusters in schools in context of community transmission.

### Ethics

The use of anonymized and aggregated surveillance data does not require ethical approval and consent to participate. Use of individual level data was carried out in accordance with national guidelines and regulations. Access to the Norwegian individual level data was provided according to the Health Preparedness Act § 2–4 and permitted by the Norwegian Regional Committee for Research Ethics (REK Sør-Øst A, ref. 198,964). The need for informed consent was waived by the ethics committee. According to Danish regulation, national surveillance activities including studies relying solely on registries, do not require individual consent nor approval from an ethics committee.

## Results

### Infection prevention and control measures, testing strategies and vaccination of adolescents

There were slight variations between the countries regarding existing IPC measures, testing strategies and vaccination start-up among adolescents (Table 2).

**Table 2:** Overview of IPC measures, testing- and vaccination strategies for school-aged children and adolescents across the Nordic countries, autumn 2021 (week 30–41, July 26th – October 17th)

	Denmark	Finland	Iceland	Norway	Sweden
**All measures in society lifted**	Sep 11th (regional mitigation measures may exist)	Sep 30th (regional mitigation measures may exist)	No decision	Sep 25th (regional mitigation measures may exist)	Sep 29th (mitigation measures remain unchanged for unvaccinated individuals)
**Start of universal vaccination 16–17 y**	Week 23 (June 7th)	Week 25 (June 21st)	Week 24 (June 14th)	Week 34 (Aug 23rd)	Week 29 (July 19th)
**Start of universal vaccination 12–15 y**	Week 29 (July 19th)	Week 32 (Aug 9th)	Week 26^a^ (June 28th)	Week 36 (Sep 6th) (Only one dose)	Week 42^b^ (Oct 18th)
**Testing, isolation, contact**^c^ **tracing and quarantine**	In place	In place	In place	In place	In place
	General population: Test (2 tests) and quarantine (if unvaccinated) until result of test on day 4.	General population: Quarantine for close contacts, depending on vaccination status.	General population: Quarantine for close contacts, depending on vaccination status.	General population: Quarantine for close contacts, depending on vaccination status.	General population: Quarantine and screening for unvaccinated household and other close contacts.
	School (from Sep 9th)^d^: Primary and lower secondary school: test-to-stay strategy of entire class and other close contacts to replace quarantine (2–3^e^ tests)	School: Quarantine and testing of classes and other close contacts (test result did not affect quarantine practice).	School: Test-to-stay for lesser exposures (2 tests)^f^	School: Test-to-stay strategy. (2–3^e^ tests or regular testing) (except from unvaccinated household contacts).	School: Testing of classes and other close contacts. No quarantine.
**Indication for testing**	Symptomatic testing (regardless of vaccination status) Testing of household- and other close contacts Weekly testing recommended for unvaccinated ≥9 years (from Sep 9th)	Symptomatic testing^g^. Testing of household- and other close contacts (including school exposures)	Symptomatic testing. Testing of household- and other close contacts Testing in schools for larger gatherings (school events)	Symptomatic testing (regardless of vaccination status). Testing of household- and other close contacts Weekly testing in areas/ schools with high incidence/ outbreaks	Symptomatic testing > 6 years (regardless of vaccination status). Testing of household- and other close contacts > 6 years.
**IPC measures in schools**	General IPC measures (hygiene and stay at home if sick policy)	General IPC measures (hygiene and stay at home if sick policy)	General IPC measures (hygiene and stay at home if sick policy)	General IPC measures (hygiene and stay at home if sick policy)	General IPC measures (hygiene and stay at home if sick policy)
	No facemasks	Face masks for those 12 years and above^g^.	Facemasks and distancing partly in use^h^	No facemasks	No facemasks
	No cohorting or distance requirements for students	No cohorting or distance requirements for students	No cohorting in school	No cohorting or distance requirements for students	No cohorting or distance requirements for students

^a^Open for vaccination of 12–15- year-olds from week 26, but systematically started week 33/34

^b^Not initiated during study period

^c^Small variations between countries in definition of close contact

^d^Before Sep 9th: Close contacts in class/school and household contacts: PCR test on day 4 and 6 from last exposure and quarantine (if unvaccinated) until result of test on day 4

^e^Number of tests recommended depending on vaccination status

^f^If 1 case in a class; only close contacts in quarantine and test-to-stay strategy for the remaining students (2 tests; day 0 and day 4). If  ≥2 cases in same class within 14 days, then quarantine of the class

^g^Recommendations changed week 41 regarding testing (no testing for those with mild symptoms and fully vaccinated/children < 12, if not exposed), and face mask use (lifted at schools)

^h^Face masks and distancing recommended for older students (children ≥12 years) during limited periods

All countries except Iceland lifted the general mitigation measures in society towards the end of the study period. In schools, all countries recommended basic IPC measures, e.g. hygiene and stay-home-when-sick policy, but no countries practiced cohorting. Only Iceland had some distancing recommendations for the older students. Face masks were only used by older students (≥12 years old) in Finland and in Iceland (for limited periods). The indication for testing varied slightly between the countries, but all recommended symptomatic testing and testing of both household- and other close contacts. Denmark, Iceland and Norway used test-to-stay strategy for students in schools. Denmark and Norway also implemented weekly testing/ screening; in Denmark this applied to all unvaccinated students ≥9 years old, while in Norway this was only implemented in schools or areas with high incidence rates, and mostly for students ≥10 years old.

Vaccination for the 16–17-year-olds was first initiated in Denmark (from week 23 (Jun 7th), followed by Iceland and Finland (week 24 and 25 (Jun 14th and 21st), respectively). For the age group 12–15 years, Iceland and Denmark initiated vaccination before the study period started (week 26 and 29 (Jun 28th, Jul 19th), respectively). Children < 12 years were not offered vaccination in any of the countries during the study period.

### Infection rates in students and vaccine uptake in the population

**Fig. 1:**
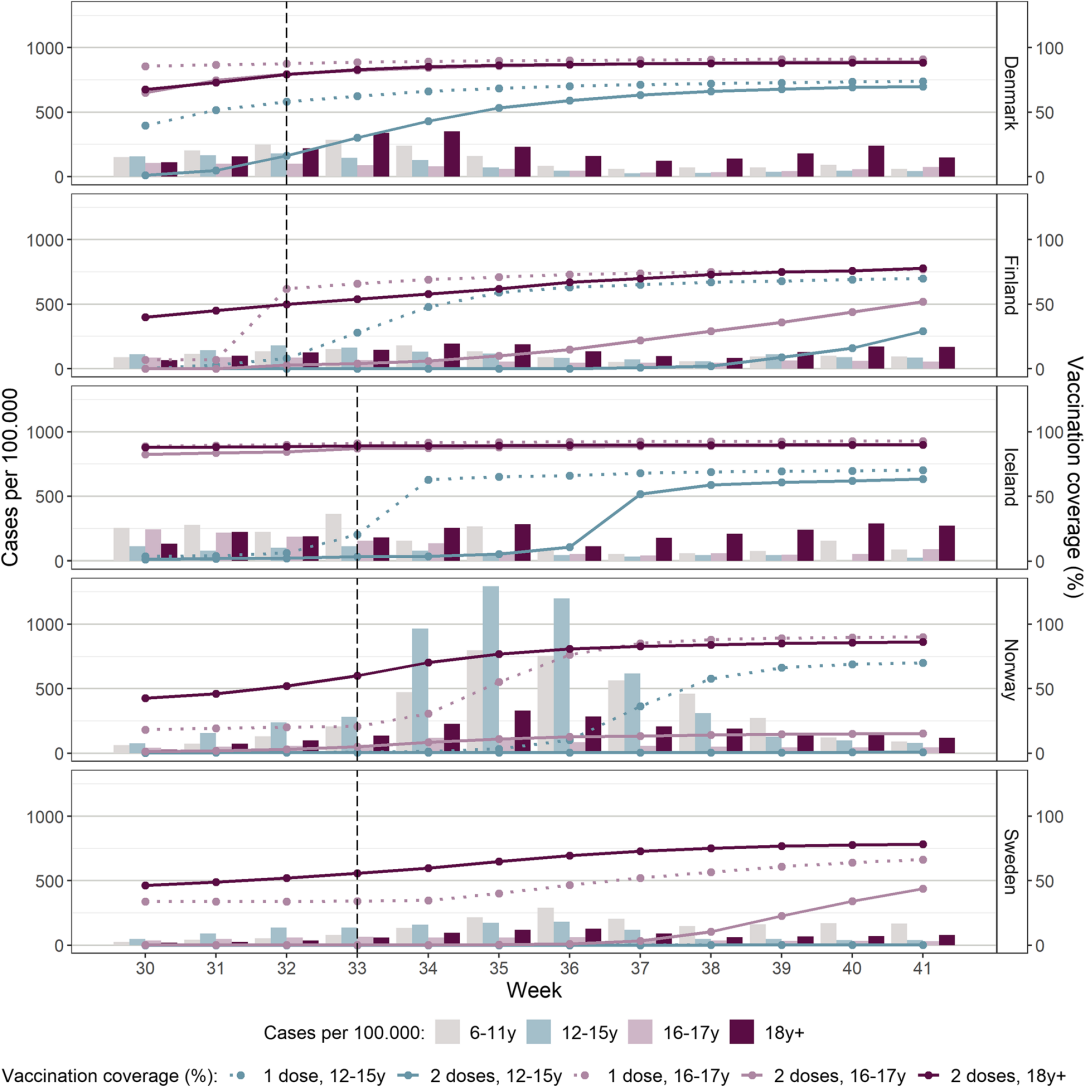
Incidence and vaccine uptake for each country, week 30–41 (Jul 26th – Oct 17th), 2021. Weekly number of cases per 100.000 (bar) and vaccine uptake (line), stratified by age groups (6–11, 12–15 and 16–17 years) according to vaccination priority. The figure also includes vaccine uptake (for dose 2) in adults (≥18 years). Black vertical line illustrates week of school start

**Fig. 2:**
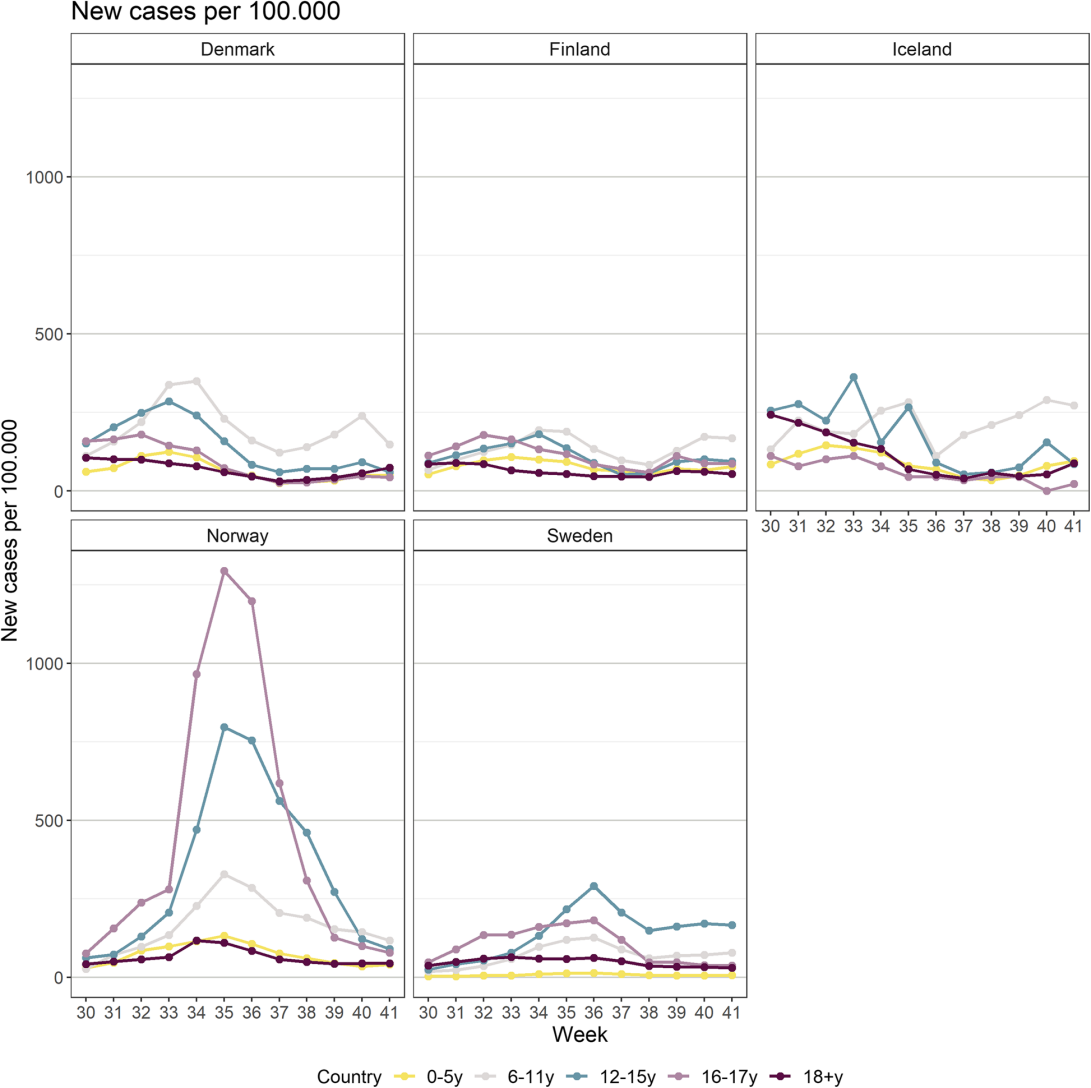
Incidence rate (new cases per 100.000) stratified for different age groups between week 30–41 (Jul 26th – Oct 17th), 2021 in the Nordic countries

Population size by age group for each country is presented in Table 3. Denmark, Sweden and Norway observed a temporary rise in incidence in the school-aged population (6–17 years) following reopening of schools (Fig. 1). Norway experienced the highest incidence rates, especially among 16–17-year-olds. The peak coincided with the weeks before vaccination started for this age group. In Iceland, a surge was already on the way when schools reopened (week 33), but no overall increase was observed after schools resumed. In Finland, only small variations were observed during the period. Furthermore, Denmark, Iceland and, to a lesser extent, Finland experienced an increase in the number of cases in the age group 6–11 years (unvaccinated) towards the end of the study period. The incidence in the adult population (≥18 years) was very low in all countries, except from the surge in Iceland. Infection incidence for the whole population (age groups: 0–5, 6–11, 12–15, 16–17, ≥18 years) is presented in Fig. 2.

**Table 3:** Population size by age group for each country

	0–5 y	6–11 y	12–15 y	16–17 y	18+ y	Total
Finland	314,157	352,334	249,816	121,445	4,528,035	5,565,787
Norway	343,764	382,451	258,632	126,843	4,279,679	5,391,369
Sweden	718,219	751,189	486,308	233,687	8,189,892	10,379,295
Denmark	372,243	369,573	272,889	136,694	4,698,790	5,850,189
Iceland	26,152	28,596	18,746	8942	286,356	368,792

All countries had high vaccination coverage for dose 2 in the population ≥ 18 years, increasing during the study period to 90% (Iceland), 89% (Denmark), 86% (Norway) and 78% (Finland and Sweden) (Fig. 1). The uptake for dose 1 and dose 2 in adolescents varied between countries. In Denmark and Iceland, the uptake in 16–17-year-olds was close to that of the adult population at the start of the school year. Finland early initiated dose 1 for both age groups, but had lower coverage for dose 2 than Iceland and Denmark. Norway and Sweden had the lowest vaccination coverage among adolescents. In Norway, vaccination for 16–17-year-olds started after the school opened and dose 2 was not yet provided during the study period. Vaccination of the 12–15-year-olds had the latest start-up in all countries and the largest variation in uptake between countries. Sweden did not initiate universal vaccination for 12–15-year-olds during the study period, while Norway did not offer the second dose for this age group.

### Infection clusters in school-aged children (Denmark and Norway)

**Fig. 3:**
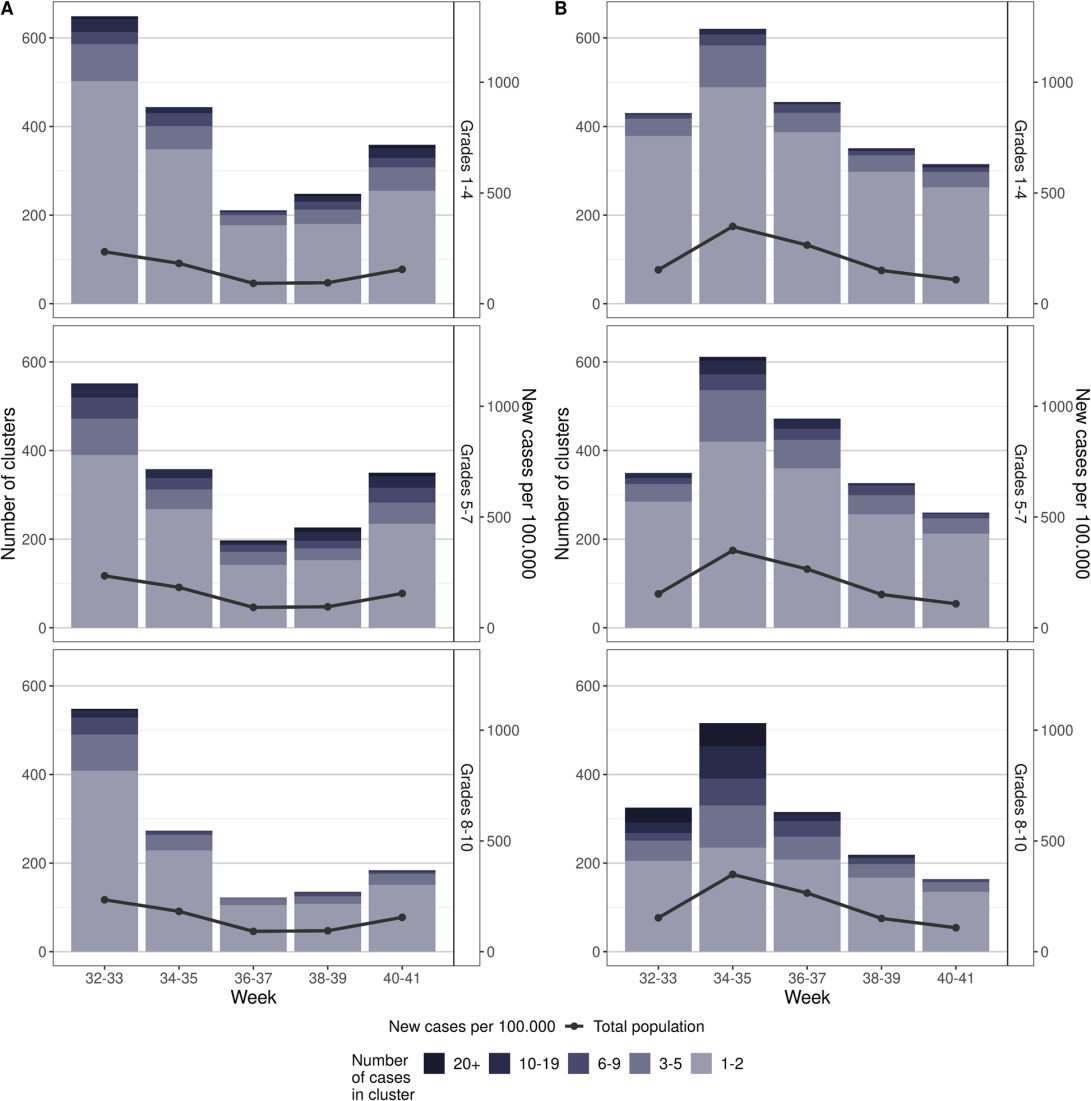
Number and distribution of new clusters in Denmark (A) and Norway (B) per two-week periods, stratified by cluster size, and incidence (new cases per 100.000) in the total population. Week 32–41 (Aug 9th – Oct 17th), 2021

The number of clusters per week reflected the incidence trend of the country (Fig. 3). For both countries most clusters (75%) consisted of 1–2 cases (sporadic cases). In Norway, sporadic cases were more common in lower than higher grades, but more evenly distributed among school grades in Denmark (Table 4). The larger clusters (≥10 cases) were most frequent in higher grades in Norway (grades 8–10) as opposed to in Denmark, where these clusters were more frequent in lower grades (grades 1–7). In both countries, large clusters were more common in periods with high incidence in the general population.

**Table 4:** Number (%) of new clusters stratified by school grades and cluster size

Cluster size	Denmark			Norway		
	Grades 1–4^a^	Grades 5–7	Grades 8–10	Grades 1–4	Grades 5–7	Grades 8–10
**1–2**	1463 (76.6)	1188 (70.6)	1002 (79.4)	1816 (83.6)	1534 (76.0)	950 (61.7)
**3–5**	244 (12.8)	229 (13.6)	174 (15.6)	249 (11.5)	296 (14.7)	246 (16.0)
**6–9**	103 (5.4)	140 (8.3)	62 (4.9)	73 (3.4)	108 (5.4)	133 (8.6)
**10–19**	82 (4.3)	99 (5.9)	21 (1.7)	28 (1.3)	70 (3.5)	120 (7.8)
**20+**	19 (1.0)	26 (1.6)	3 (0.2)	5 (0.2)	10 (0.5)	90 (5.8)
**Total**	**1911 (100.0)**	**1682 (100.0)**	**1262 (100.0)**	**2171 (100.0)**	**2018 (100.0)**	**1539 (100.0)**

^a^Approximate age ranges for the grades: Grade 1–4: 6–9 years, grade 5–7: 10–12 years, grade 8–10: 13–15 years

## Discussion

We here present trends in SARS-CoV-2 infections among children and adolescents in school during the first months of the autumn semester 2021 in the Nordic countries, when the SARS-CoV-2 Delta variant dominated. Schools opened for full in-person teaching in all countries, with moderate IPC measures. Our study showed that after schools opened, infection incidence and trends in the younger age groups varied between countries. Several factors, i.e. vaccination strategy for adolescents, testing activity and other mitigation measures in and outside school, may have influenced these differences.

The low incidence observed in adolescents (12–15 and 16–17-years) in Denmark and Iceland, may in part be explained by the high vaccination coverage in these age groups when schools opened. In Finland, dose 1 was initiated early for both age groups, but coverage for dose 2 was lower than in Iceland and Denmark due to a prolonged interval between the doses. Despite this, the incidence was low in all age groups in Finland throughout the study period. Norway and Sweden had the lowest vaccination coverage among adolescents. However, the infection incidence in this group was much higher in Norway than in Sweden. This may partly be explained by higher natural immunity (seropositivity) in Sweden at the time [37] and/or systematic screening in Norway.

Although the evidence of effect is limited, test-to-stay strategies for school contacts have been implemented as an alternative to quarantine in order to reduce secondary transmission in schools while increasing in-person learning [38–41]. This strategy was used by some of the Nordic countries (Denmark, Iceland and Norway). In addition, Denmark and Norway initiated weekly screening in schools within the first few weeks of the semester. This screening probably contributed to detection of a higher number of cases, which may also in part explain the differences in incidence observed among adolescents in Norway and Sweden. However, although Finland and Sweden did not perform regular screening, extensive testing around cases in schools was performed in both countries. This suggests that the difference in incidence at that time between countries can be considered reliable. Extensive screening, even when done at home with self-tests, also implies a burden and may cause stress and discomfort for children. Given the low risk for severe disease due to COVID-19 in children [2–6], use of testing strategies should be carefully considered and limited.

Results based on data from surveillance systems of clusters in schools in Denmark and Norway revealed that the majority of index cases did not result in further transmission in schools. This is in line with previous studies [10, 33, 34, 42–46], and underlines findings that schools were less important arenas for secondary transmission compared with e.g. households [10, 47, 48]. Indeed, transmission in schools seems to reflect community infection rates, as both size and number of clusters detected in schools increased during periods with higher community infection rates. This is consistent with observations from other studies [34, 49]. Still, we observed some differences between Denmark and Norway. Larger clusters were more common in older age groups in Norway, but in younger age groups in Denmark. This may relate to different vaccination and testing strategies, as discussed. The high vaccine uptake for adolescents in Denmark at the time may have reduced the cluster sizes in this age group.

Experiences from the Nordic countries indicate that vaccination of adolescents was followed by reduced infection incidence in the respective age groups. The incidence in the youngest age group (6–11 years) was relatively similar in all countries. Along with increasing vaccination coverage in the older age groups, we observed a slight increase in incidence in 6–11-year-olds towards the end of the study period. This was most pronounced in Denmark and Iceland, which had the overall highest vaccination coverage in all other age groups. With higher vaccine uptake in the general population, higher infection incidence is expected in unvaccinated population groups. This was indeed seen in all the Nordic countries following the study period, when a rapid increase in incidence was observed in the unvaccinated age groups in the remaining months of the fall semester [27–31].

Overall, we observed that incidence peaks in student age groups could be reversed even with schools open for in-person teaching and with moderate mitigation measures in place. High vaccine uptake in the adult population and among adolescents, as well as active testing strategies followed by isolation of cases, may have been contributing factors. This observation is in line with findings from other studies [45, 46].

### Limitations

Our observations are from a period dominated by the Delta variant and may therefore not be directly transferrable to periods with other circulating virus variants. Following the introduction of the more transmissible Omicron variant, incidence rose sharply in all age groups in all countries [27–31]. However, current knowledge indicates that this variant is less virulent than previous variants [44, 50, 51], and therefore mitigation measures should be adjusted according to the total burden of diseasee. Another limitation is that the register-based school cluster surveillance only identified clusters in time and place and cannot separate between transmission in schools and transmission in the community. This may result in artificially large school clusters in periods with high community transmission, due to the probability of multiple separate introductions in each school and age cohort. The Nordic countries have largely similar structures and societies, but even small variations may challenge direct comparison of results between the countries. For instance, comparison of incidence in children versus adolescents and adults is influenced by different vaccination status and different testing strategies in these groups. Also, as this is an observational study, it is not possible to analyze the effects of IPC measures or vaccination on SARS-CoV-2 transmission. However, as it has not been possible to conduct large, randomized studies on effect of measures directed towards children and adolescents in the Nordic countries during this period, evidence from observational studies may provide the best available knowledge.

### Public health implications

The Nordic countries are quite homogenous regarding education systems, surveillance systems, vaccination programs, testing strategies and mitigation measures. However, due to differences in societal and structural factors, our observations may not be directly transferrable to other countries and regions. Still, our findings add to the growing evidence on incidence and transmission of COVID-19 in educational settings and may be valuable for decision makers worldwide.

COVID-19 continues to spread globally. While most countries have now lifted COVID-19 specific mitigation measures in schools, it is becoming clear that closures and strict measures had negative short- and long-term consequences for children’s learning and quality of life [15, 18–25] Students have had vastly different opportunities to participate in digital classes, which has led to enhanced inequality and larger educational delays among children with disadvantaged backgrounds [9, 52]. Our observations support that full in-person teaching is possible also in periods with relatively high infection rates, when vaccination coverage in the general population is high and targeted mitigation measures are in place. Continued surveillance and research in this field is of great importance to secure education for children and adolescents around the world.

## Conclusion

Children and adolescents have a right to attend school. In-person education is vital both for learning and for their health and wellbeing. Keeping schools open should therefore be a priority. Data from the Nordic countries indicate that vaccination of adults and adolescents, in addition to moderate mitigation measures, may be important factors to achieve this goal. As SARS-CoV-2 infection does not represent a severe medical risk for most children, measures should be carefully adjusted and reduced to a minimum to ease their burden.

## Data Availability

Most of the data used and/or analysed during the current study are available on the national institutes’ websites (listed in Table 1). Additional data available from the respective national institutions, via the corresponding author, on reasonable request.
